# Synergistic Photoantimicrobial Chemotherapy of Methylene Blue-Encapsulated Chitosan on Biofilm-Contaminated Titanium

**DOI:** 10.3390/ph14040346

**Published:** 2021-04-09

**Authors:** Chiu-Nan Lin, Shinn-Jyh Ding, Chun-Cheng Chen

**Affiliations:** 1 Institute of Oral Science, Chung Shan Medical University, Taichung City 402, Taiwan; charliennnn@gmail.com; 2 Department of Stomatology, Chung Shan Medical University Hospital, Taichung City 402, Taiwan; 3 School of Dentistry, Chung Shan Medical University, Taichung City 402, Taiwan

**Keywords:** dental implant, peri-implantitis, photoantimicrobial chemotherapy, methylene blue, chitosan

## Abstract

Intensive efforts have been made to eliminate or substantial reduce bacterial adhesion and biofilm formation on titanium implants. However, in the management of peri-implantitis, the methylene blue (MB) photosensitizer commonly used in photoantimicrobial chemotherapy (PACT) is limited to a low retention on the implant surface. The purpose of this study was to assess enhancive effect of water-soluble quaternary ammonium chitosan (QTS) on MB retention on biofilm-infected SLA (sandblasted, large grid, and acid-etched) Ti alloy surfaces in vitro. The effectiveness of QTS + MB with different concentrations in eliminating Gram-negative *A. actinomycetemcomitans* or Gram-positive *S. mutans* bacteria was compared before and after PACT. Bacterial counting and lipopolysaccharide (LPS) detection were examined, and then the growth of human osteoblast-like MG63 cells was evaluated. The results indicated that the synergistic QTS + MB with retention ability significantly decreased the biofilm accumulation on the Ti alloy surface, which was better than the same concentration of 1 wt% methyl cellulose (MC). More importantly, the osteogenic activity of MG63 cells on the disinfected sample treated by QTS + MB-PACT modality was comparable to that of sterile Ti control, significantly higher than that by MC + MB-PACT modality. It is concluded that, in terms of improved retention efficacy, effective bacteria eradication, and enhanced cell growth, synergistically, PACT using the 100 μg/mL MB-encapsulated 1% QTS was a promising modality for the treatment of peri-implantitis.

## 1. Introduction

When the pristine surface of a dental titanium implant is exposed to the oral cavity after installation, bacterial adhesion occurs in a similar way to teeth [1,2]. After adhesion, bacteria will form microcolonies, which are then embedded within an extracellular polysaccharide matrix to construct complex three-dimensional biofilm structures that are difficult to remove [2,3]. More importantly, bacterial contamination endangers the osseointegration of titanium implants [4,5] and ultimately leads to bone loss around the implant. According to the literature [6], after 10 years of use without systematic supportive treatment, peri-implant disease has become a common clinical problem. Van Velzen et al. evaluated the 10-year survival rate of SLA (sandblasting, large-grit and acid-etching) titanium dental implants and the incidence of peri-implant diseases in patients with complete and partial edentulousness, and pointed out the occurrence of peri-implantitis in 7% of dental implants [7]. As the prevalence of peri-implantitis has increased significantly, as many as 33% of implants have experienced progressive bone loss [8]. Thus, in order to successfully treat peri-implantitis, exhaustive eradication of pathogenic microorganisms from the surface of the implant is indispensable [9,10,11,12]. The current clinical approach is to use mechanical debridement to destroy biofilms and reduce bacterial adhesion [13], supplemented by adjunct antibacterial therapies, such as the use of antiseptics and antibiotics [11,14,15]. However, the efficacy of these strategies is limited by the complex topography of the implant surface, the emergence of drug-resistant microorganisms, and the survival of a few viable bacteria. Therefore, the strategies used cannot completely cause the osseointegration of the implant to a large extent [16]. In fact, there is currently no gold standard method for the treatment of peri-implantitis [15]. Undoubtedly, it necessitates developing newly effective treatments targeting bacterial eradication for clinical use.

For this reason, extensive investigations have been explored to develop photodynamic therapy (PDT), which was introduced as a medical treatment in 1904 [17]. PDT uses a specific wavelength of light to activate a photosensitive drug (photosensitizer, PS) that preferentially binds to cells or microorganisms in the presence of oxygen in the air. This may lead to the formation of several reactive oxygen species, resulting in cell death [15,18]. Among various applications, such as antimicrobial elimination, anticancer therapy, and wound healing [19,20,21], the former is the so-called antimicrobial photodynamic therapy (aPDT) or photoantimicrobial chemotherapy (PACT) [22]. The PACT modality has been used alone or in combination with other treatment options, such as mechanical debridement.

As for photosensitizers, the commonly used methylene blue (MB) is a hydrophilic, water-soluble phenothiazine derivative and the first synthetic compound used as an antiseptic in clinical practice [23]. Moreover, it has been approved by the Food and Drug Administration (FDA) for intravenous administration of methemoglobinemia [24] and has been used to target oral bacteria [25]. In the management of peri-implantitis, MB-PACT treatment usually aims to eliminate bacterial adhesion and biofilm on the implant surface, thereby achieving re-osseointegration. However, satisfactory therapeutic strategies or scientifically based treatment recommendations are still not available [17,26]. For example, in terms of clinical practice, the retention capacity of the flowable photosensitizer should be considered, and the loss of photosensitizer during use should be avoided to improve the therapeutic effect. To overcome this problem, viscous polymers can be adopted to achieve the adhesion of photosensitive drug to mucosa (such as oral buccal mucosa, nasal mucosa) [27,28]. Bioadhesion strategies can increase the retention time and availability of drugs [29]. In order to improve the MB retention during PACT, López-Jiménez et al. used hydroxymethyl cellulose as a mucoadhesive in the Periowave system [30].

Among the viscous polymers, naturally occurring chitosan polysaccharides are structurally similar to glycosaminoglycans [31]. Chitosan polysaccharide has non-antigenicity, high hydrophilicity and good film-forming properties, and is a natural choice for biomedical applications (such as scaffolds and mucosal carriers) [32,33,34,35]. It has been reported that due to the interaction between the cationic chain of chitosan and the negatively charged residues on the bacterial surface, the broad antibacterial spectrum of chitosan is effective against Gram-positive and Gram-negative bacteria and fungi [36,37]. On the other hand, positively charged chitosan may be a good choice as a PS carrier and bioadhesive, which can increase the retention at the application site [38]. This is because on the basis of charge repulsion, cationic drugs will be rapidly released from the cationic hydrogel [39]. Moreover, the hydrophilic chitosan facilitates the close contact between the PS surface and the aqueous environment of microorganisms [40], which has broad prospects for the delivery of PS. Shrestha et al. pointed out that the use of chitosan-conjugated photosensitizers had a synergistic advantage over the use of photosensitizers alone, which can improve the anti-biofilm efficacy of Gram-positive bacteria and Gram-negative bacteria [40]. Choi et al. found that pretreatment with chitosan before MB-medicated PDT can effectively improve the photodynamic effect of MB at a low concentration of 40 μg/mL on the eradication of *H. pyloriat* [41]. Therefore, it is speculated that the MB encapsulation in viscous chitosan can stay on the surface of the infected implant for a long time and achieve a higher photo-inactivation efficacy against bacteria. However, the commonly used chitosan polysaccharide has the disadvantage of being only dissolved in an acidic solution, which results in a relatively low pH solution of less than 3. More importantly, from a clinical point of view, the pH of the solution is preferably 7 because of oral environment. In this regard, some water-soluble chitosan derivatives, such as quaternary ammonium chitosan (QTS), can replace acid-soluble chitosan polysaccharides [35]. Herein, we chose QTS as the adhesive (or carrier) and analyzed the antimicrobial effect of QTS + MB-PDT on Gram-positive and Gram-negative bacteria. It is hypothesized that the synergistic combination of MB-conjugated QTS (QTS + MB) could have the dual functions of enhancing the photo-inactivation effect and improving the adhesion stability of the photosensitizer, which in turn maintains the osseointegration of the implant. The overall experimental process and purpose were schematically illustrated in Figure S1.

## 2. Results

### 2.1. Cytotoxicity

**Figure 1:**
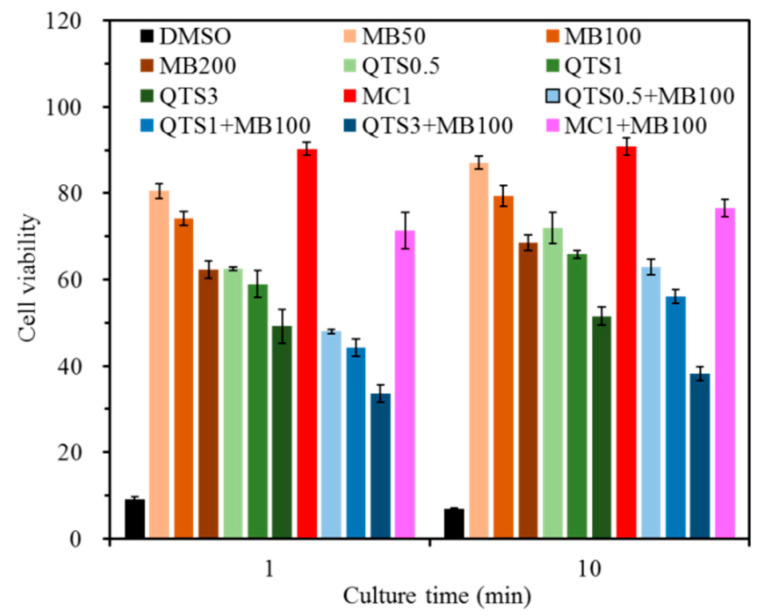
Cytotoxicity of L929 cells cultured with different test agents for 1 and 10 min. The cell viability was normalized to the negative control (culture medium) in terms of absorbance (*n* = 3).

Figure 1 shows the effects of different formulations on L929 cytotoxicity. Obviously, the viability value of the positive control (DMSO) was less than 10% at the two culture time points, and showed significant lower (*p* < 0.05) cell viability than other test samples. When cells were seeded with MB and/or QTS, a concentration-dependent decrease in cellular activity was noted. At 1 min of culture, as the concentration increased, the viability of the cells inoculated with MB alone decreased from 81% to 62%. Similar to the downward trend, the cell viability of QTS was between 63% and 49%. However, the value of L929 cells cultured with MC1 agent was higher than 90%, indicating no signs of cytotoxicity, but the co-presence of MB100 caused the viability value down to 71%. In the case of the MB100 agent with different QTS concentrations, the value was significantly (*p* < 0.05) lower than the corresponding one without MB100. After 10 min of culture, it is worth noting that, except for the positive control, the viability values of all samples increased slightly.

### 2.2. Viscosity

**Figure 2:**
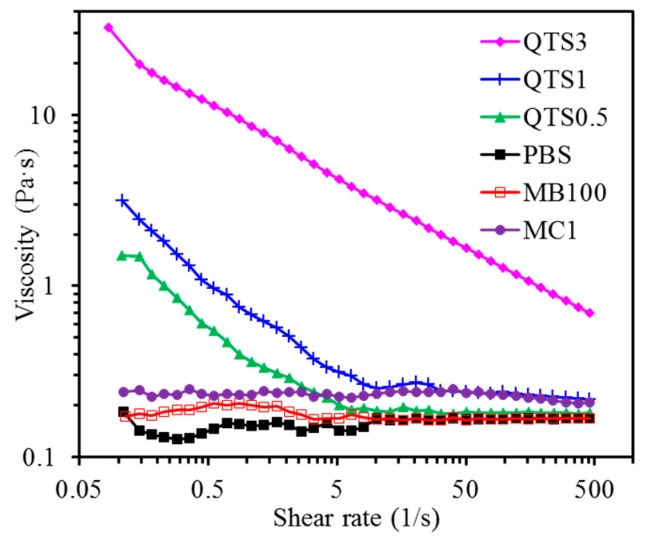
The viscosity changes of various solution samples under different shear rates.

The viscosity of various solutions varies with the shear rate, as shown in Figure 2. Phosphate buffer solution (PBS) and MB100 did not change remarkably with the increase of shear rate. When the shear rate was great than 5 1/s, their viscosity was similar. However, the viscosity of the two solutions was obviously lower than that of the three QTS and MC1 solutions. The viscosity of the three QTS solutions decreased as the shear rate increased to 5 1/s. A higher concentration of QTS caused a larger viscosity. For example, the apparent viscosities of QTS0.5 and QTS1 were about 0.20 and 0.31 Pa·s at the shear rate of 5 1/s, respectively. The constant viscosity of MC1 was about 0.23 Pa·s.

### 2.3. Retention Efficacy

**Figure 3:**
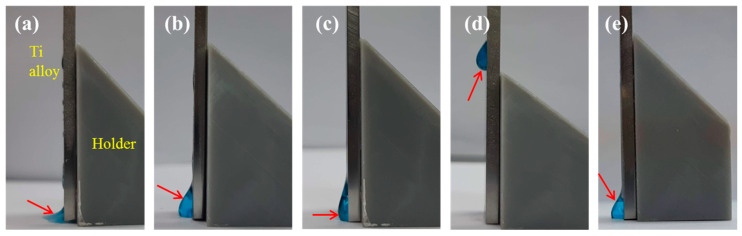
Images photographed for the retention efficacy of (**a**) methylene blue (MB)100, (**b**) quaternary ammonium chitosan (QTS)0.5 + MB100, (**c**) QTS1 + MB100, (**d**) QTS3 + MB100, and (**e**) MC1 + MB100 photosensitizers after the test solution was placed on the top of Ti alloy plate with 90-degree angle. Arrow indicates the flowing test solution.

It can be clearly seen that MB100 alone flows directly from the top to the bottom of the Ti alloy plate, as shown in Figure 3a. On the contrary, as the concentration of QTS increased, the QTS + MB solution can be retained on the inclined surface (Figure 3b–d), especially the QTS3 + MB100 droplet was completely remained on the vertical plate. A part of MC1 + MB100 solution was stuck on the plate (Figure 3e). From the perspective of the retention of the solution, the order of the viscous agent’ ability to retain MB100 was QTS3 > QTS1 > MC1 > QTS0.5.

### 2.4. Bacteriostatic Ratio

**Figure 4:**
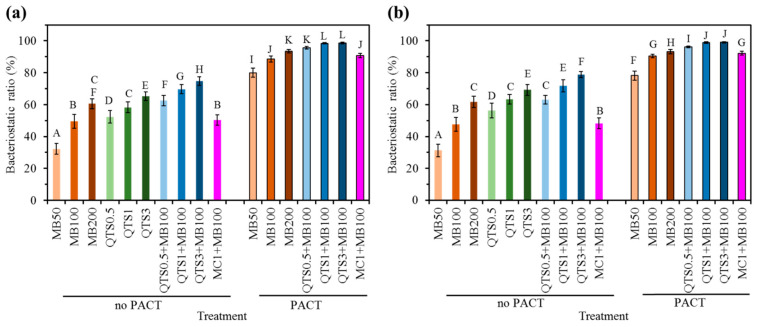
Bacteriostatic ratios of (**a**) *A. actinomycetemcomitans*-contaminated and (**b**) *S. mutans*-contaminated samples after various treatments with and without photoantimicrobial chemotherapy (PACT). Different capital letters showed statistically significant differences at *p* < 0.05 (*n* = 10).

In the absence of laser irradiation, as the concentration of MB increased, *A. actinomycetemcomitans* (Figure 4a) and *S. mutans* (Figure 4b) attached to Ti alloy surfaces were more inactivated or killed. For example, the killing amount of *A. actinomycetemcomitans* in the MB100 and MB200 groups was 49% and 60%, respectively. Similar to these findings, regardless of the type of bacteria, the QTS group showed a trend in a significantly different manner. More importantly, the combinational MB and QTS modality exhibited a synergistic killing effect, which was significantly higher (*p* < 0.05) than that of the corresponding MB alone or QTS alone. However, MC1+MB100 had the same effect as MB100. By laser irradiation, when the MB group was compared with the corresponding MB-PDT group, in addition to the concentration-dependent bacteriostatic ratio, enhanced antibacterial efficacy was also found. When using MB100-PACT, approximately 89% of *A. actinomycetemcomitans* and 91% of *S. mutans* were eliminated. It is worth noting that the bacteriostatic ratio of the three QTS+MB100-PACT groups was higher than that of the MB100-PACT and MC1 + MB-PACT groups. In addition, QTS1 + MB100-PACT and QTS3 + MB100-PACT had similar results on *A. actinomycetemcomitans* and *S. mutans*, with a bacterial death rate of over 98%.

### 2.5. Bacterial Colonies

**Figure 5:**
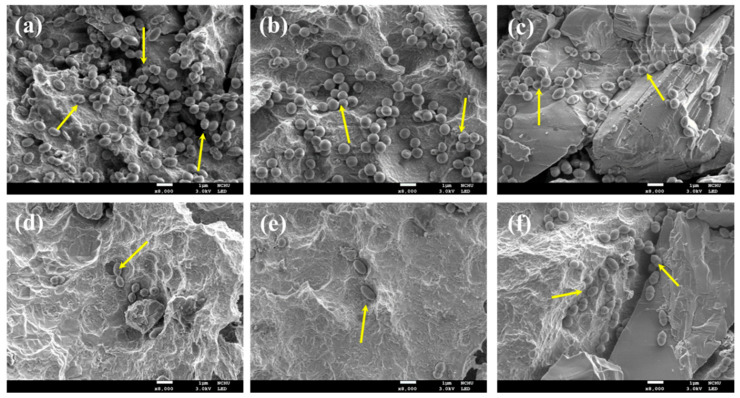
SEM images of the *A. actinomycetcmcomitans*-contaminated SLA Ti alloy surfaces (**a**) before and after treatment with (**b**) QTS1 alone, (**c**) MB100 alone, (**d**) MB100-PACT, (**e**) QTS1 + MB100-PACT, and (**f**) MC1 + MB100-PACT. The arrows indicate the presence of bacteria.

The observation of bacterial colonies on the sample surface further depicted the elimination efficacy of different treatments, including MB100, QTS1, and MC1. Before treatment (Figure 5a), a large number of oval-shaped *A. actinomycetemcomitans* colonies uniformly adhered to the contaminated surface. Upon cleaning with QTS1 alone (Figure 5b) or MB100 alone (Figure 5c), the number of bacteria was reduced to some degree. Furthermore, the number of *A. actinomycetemcomitans* colonies on the decontaminated surface treated by MB100-PACT (Figure 5d) and MC1 + MB100 (Figure 5f) surfaces was remarkably lower than the number on the contaminated surface. Not surprisingly, the QTS1 + MB100-PACT treatment resulted in a sparse distribution of colonies (Figure 5e).

**Figure 6:**
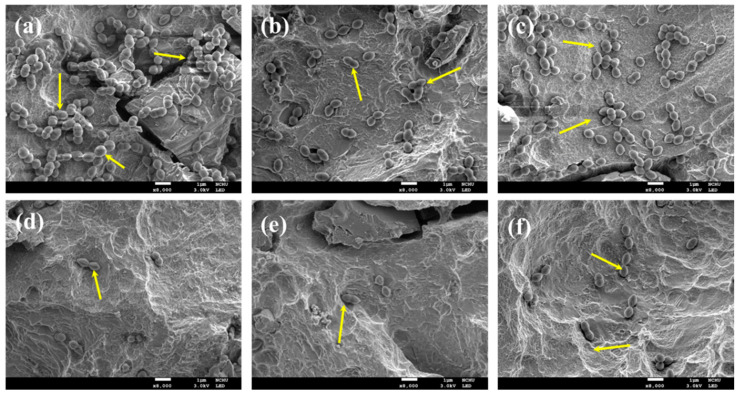
SEM images of the *S. mutans*-contaminated SLA Ti alloy surfaces (**a**) before and after treatment with (**b**) QTS1 alone, (**c**) MB100 alone, (**d**) MB100-PACT, (**e**) QTS1 + MB100-PACT, and (**f**) MC1 + MB100-PACT. The arrows indicated the presence of bacteria. The arrows indicate the presence of bacteria.

Regarding the colonies of *S. aureus*, densely packed sphere-like microcolonies were shown on the surface of the biofilm-contaminated samples (Figure 6a). Similar to the results of A. actinomycetemcomitans-contaminated surface, the treatment by QTS1 alone (Figure 6b) and MB100 alone (Figure 6c) reduced the number of colonies on the sample surfaces. In contrast, through various PDT modalities (Figure 6d–f), the number of bacterial colony-forming units (CFU) was greatly reduced when compared with the control and solution groups alone.

### 2.6. Residual LPS Amount

**Figure 7:**
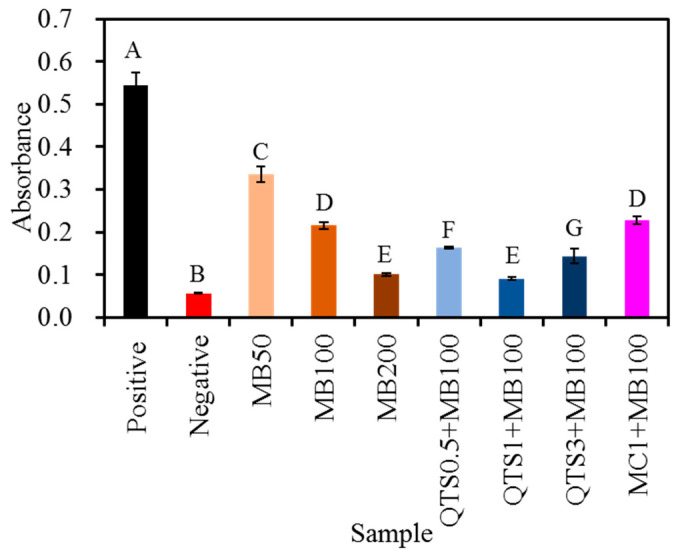
Residual lipopolysaccharide (LPS) level derived from *A. actinomycetcmcomitans* on sample surfaces after various photoantimicrobial chemotherapy (PACT) treatments. The contaminated Ti alloy without PACT and the sterile Ti alloy were used as a positive control and negative control, respectively. Different capital letters showed statistically significant differences at *p* < 0.05 (*n* = 5).

The effect of PACT treatment on the amount of LPS derived from *A. actinomycetcmcomitans* remaining on the sample surfaces is shown in Figure 7. There was no doubt that the LPS amount in the contaminated Ti control was higher. Conversely, the LPS amount remaining on various MB-PACT-treated surfaces was significantly (*p* < 0.05) reduced. The higher the MB concentration in the PACT treatment, the lower the residual amount of LPS exhibited. When using QTS to encapsulate MB, it was obvious that the synergetic QTS + MB100-PACT modality diminished the amount of LPS, more than the corresponding MB100-PACT group, especially QTS1+MB100-PACT. Compared with the MB100-PACT group, incorporation of 0.5%, 1%, and 3% QTS significantly (*p* < 0.05) reduced the residual amount of LPS by 24%, 57%, and 33%, respectively. However, there was no significant (*p* > 0.05) difference between MB100-PACT and MC1+MB-PACT.

### 2.7. Cell Morphology

**Figure 8:**
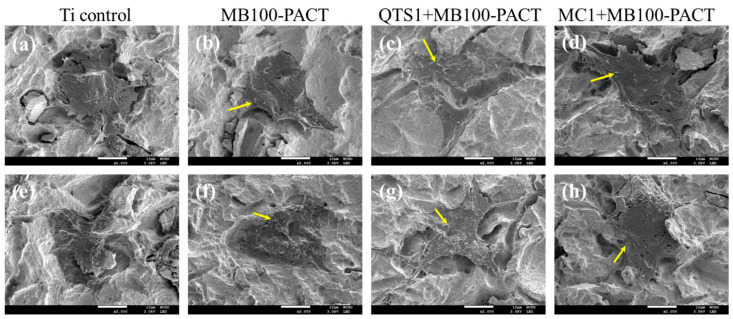
SEM images of MG63 cells after 6 h of culture on the (**a**,**e**) sterile Ti surface, (**b**–**d**) *A. actinomycetcmcomitans*-contaminated, or (**f**–**h**) *S. mutants*-contaminated surfaces treated by (**b**,**f**) MB100-PACT, (**c**,**g**) QTS1 + MB100-PACT, and (**d**,**h**) MC1 + MB100-PACT.

The initial cell morphology attached to the disinfected surfaces of samples treated with MB100-PACT, QTS1 + MB100-PACT, and MC1 + MB100-PACT is shown in Figure 8, in addition to the sterile Ti alloy (Figure 8a,e). The cells on the surface of the uncontaminated Ti alloy were well adhered and fully spread with a well-defined morphology after 6 h of culture. When MG63 cells were cultured on surfaces treated with MB100-PACT (Figure 8b,f) and MC1 + MB-PACT (Figure 8d,h), it seemed that cell attachment was adversely affected, showing less spreading. In the case of QTS1+MB100-PACT (Figure 8c,g), MG63 cells adhered well to the decontaminated surface, indicating the extending filopodia. Nevertheless, there were still a few bacteria on all decontaminated surfaces.

### 2.8. Cell Proliferation

**Figure 9:**
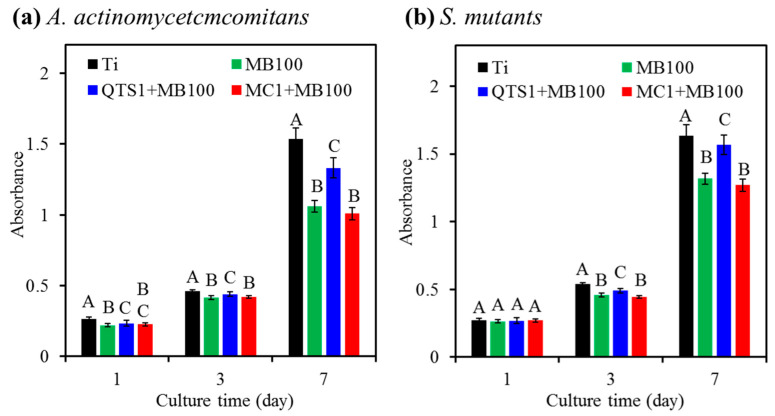
The cell viability was normalized to the negative control (culture medium) in terms of absorbance (*n* = 3). Proliferation analysis of MG63 cells cultured on the surfaces after PACT (MB100, QTS1 + MB100 and MC1 + MB100) treated samples contaminated with (**a**) *A. actinomycetcmcomitans* and (**b**) *S. mutants*. The sterile Ti alloy was used as a control. Different capital letters showed statistically significant differences at *p* < 0.05 (*n* = 5).

After culturing MG63 cells on PACT-decontaminated sample surfaces and the sterile Ti alloy surfaces, the number of viable cells increased during the incubation interval due to the increase in absorbance (Figure 9). Regarding the samples infected with *A. actinomycetcmcomitans*, the cell proliferation of all PDT-treated groups was significantly (*p* < 0.05) lower than that of the sterile Ti control (Figure 9a). On the 7th day, the cell proliferation of the QTS1 + MB100 group was significantly (*p* < 0.05) higher than that of the MB100 and MC1 + MB100 groups. For samples contaminated with *S. mutants*, the proliferation of MG63 cells in the sterile Ti control was also higher than that of all PACT groups (Figure 9b). In addition, the QTS1+MB100 group was superior to the MC1+MB100 group except for one-day culture.

### 2.9. Alkaline Phosphatase (ALP) Activity

**Figure 10:**
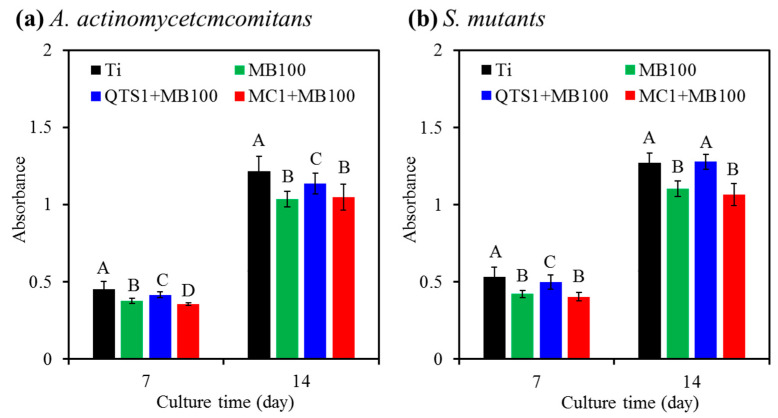
ALP activity of MG63 cells cultured on the surfaces after PACT (MB100, QTS1 + MB100 and MC1 + MB100) treated samples contaminated with (**a**) *A. actinomycetcmcomitans* and (**b**) *S. mutants*. The sterile Ti alloy was used as a control. Different capital letters showed statistically significant differences at *p* < 0.05 (*n* = 5).

After 7 days and 14 days of culture, the ALP activity of MG63 cells on the PACT-treated samples is shown in Figure 10. The sterile Ti alloy control had the highest ALP expression than all PACT treatments against the two bacteria at all culture intervals, except for the QTS1 + MB100 group against *S. mutants* on day 14. Compared with MC1+MB100 and MB100, PACT treatment with QTS1 + MB1 produced a significantly (*p* < 0.05) higher ALP amount. On the *A. actinomycetcmcomitans*-disinfected surface for 14 days of cell culture, the ALP amount in the QTS+MB100-PACT modality was 9% higher than that of the MC1 + MB100-PACT modality (Figure 10a), which was 20% on the *S. mutants*-disinfected surface (Figure 10b).

### 2.10. Mineralization

**Figure 11:**
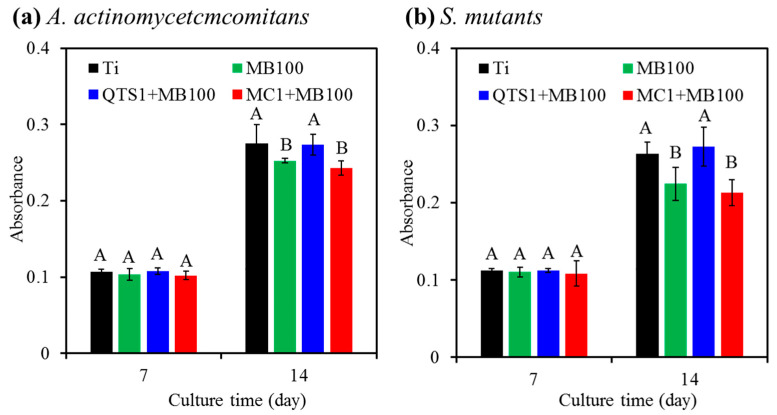
Mineralization analysis of MG63 cells cultured on the surfaces after PACT (MB100, QTS1 + MB100 and MC1 + MB100) treated samples contaminated with (**a**) *A. actinomycetcmcomitans* and (**b**) *S. mutants*. The sterile Ti alloy was used as a control. Different capital letters showed statistically significant differences at *p* < 0.05 (*n* = 5).

The results of calcium deposits secreted by MG63 cells on the biofilm-disinfected surfaces treated by different PACT modalities are shown in Figure 11, indicating the increased Ca amount with the increasing culture time. After 7 days of culture, there was no significant difference (*p* > 0.05) between all groups including the sterile Ti alloy control. On the contrary, on the 14th day, it is worth noting that the MG63 cells grown on the sterile control surface and biofilm-disinfected surface treated with QTS1 + MB100-PACT secreted a similar content of calcium deposits, and there was no significant difference (*p* > 0.05). On the other hand, at the same MB100 concentration of MB100, QTS1 agent could significantly (*p* < 0.05) cause higher Ca deposits than MC1 agent. The cells on the *A. actinomycetcmcomitans*-disinfected surface by QTS + MB100-PACT increased by about 13% compared with the MC1+MB100-PACT treatment (Figure 11a), while the cells on *S. mutants*-disinfected surface increased by 28% (Figure 11b).

## 3. Discussion

Although dental implant advancements have aimed to facilitate bone healing, the prevention of bacterial infections is also a critical factor in the success of a surgical treatment [41]. Implant-associated infections produce serious post-surgical problems that adversely affect osseointegration and result in implant device failures. Therefore, the prevention and elimination of bacterial adhesion and colonization plays an important role in the successful implantation. PACT has been considered as a reliable adjunct to conventional therapeutic modalities, such as mechanical (curettage and root planing) and surgical (transplant) methods, or can be used alone in the peri-implantitis treatment [5,42]. PACT neither causes any resistance to microorganisms, nor is affected by the resistance of existing drugs [43]. However, effective removal of biofilm and bacterial toxins from the surface of infected implants is still an unresolved clinical issue. Many studies intend to develop effective modality to eradicate bacterial colonies and biofilms on the surface of implants for restoring osseointegration.

The antimicrobial efficacy of PACT is based on the large accumulation of PS in or on the cytoplasmic membrane [44,45]. Based on clinical need, PS should have sufficient retention on the surface of infected implant for intracellular uptake, thereby progressing photo-inactivation. However, due to its fluid characteristics can lead to ineffective therapy, the retention of PS remained to be resolved. Modifying photosensitizers with viscous biopolymers will be an attractive strategy to solve this problem. Indeed, as evidenced in this study, by incorporation of a viscosity-enhancing agent, such as MC, MB retention can be given to the peri-implantitis area. MC is a water-soluble derivative of polysaccharide cellulose, and it is also a non-toxic, biocompatible FDA-approved material [46]. The introduction of viscous QTS molecules as adhesive promoters in this study can also effectively overcome the shortcomings of MB retention. This antibacterial carrier may be easily applicable to large or complex implant surfaces, and may adhere to the material substrate (or teeth). One of the current purposes was to synergistically combine MB with antibacterial and viscous QTS to construct a new type of photosensitizing platform, whereby the QTS+MB-PACT modality can effectively treat peri-implantitis.

In order to understand the concentration effect, water-dissolvable QTS at concentrations of 0.5, 1, and 3 wt% were used. The viscosity of the three QTS and MC solutions was significantly higher than that of MB. Not surprisingly, the viscosity of QTS3 was higher than QTS1 and QTS0.5. As expected, the addition of QTS1 and QTS3 effectively improve the retention of MB on the implant surface due to the viscosity effect, binding the MB together like glue. On the other hand, the viscous MC1 also kept MB part on the surface of the Ti alloy sample. However, Ludmila et al. pointed out that, in addition to preferential accumulation in bacterial targets, ideal PS should also be eliminated quickly after administration [47]. Therefore, high-viscosity QTS, such as 3% may not be suitable for use because it was difficult to remove. The long-term retention of QTS + MB on the implant surface may affect periodontal tissues and cells, resulting in the cytotoxicity [21], as described below.

Low toxicity is one of the essential characteristics of ideal photosensitizers, especially towards mammalian cells [15,48]. Therefore, when the photosensitizer was conjugated with polymers, the dose-dependent cytotoxicity of the modified PS should be examined to verify its bio-safety. From a clinical point of view, a short interval (a few minutes) in the PACT treatment of peri-implantitis was commonly employed, so this study used 1 min and 10 min of culture time. The cell viability of 50 and 100 μg/mL MB and MC1 with and without MB were all higher than 70%, while other test agents were all lower than 70%. According to ISO 10993-5 standard definition, more than 70% viability is considered non-cytotoxic [49]. Tate et al. pointed out that MC concentration as high as 8% will not cause the death of primary rat cortical astrocytes [50]. Rolim et al. reported that MB concentrations exceeding 163.5 μM (equivalent to 52.3 μg/mL) can induce cytotoxicity [51]. It is reasonable to consider that the higher the concentration of MB and QTS, the lower the cell viability, indicating that its potential cytotoxicity was higher [52,53]. Regardless of QTS or MC mucoadhesive, the combination with MB100 significantly reduced cell viability. Soukos and Goodson suggested that due to incomplete penetration of MB in oral biofilms in a clinical setting, PACT may take up to 15 min to proceed [15]. Therefore, to safely apply PS in the treatment of peri-implantitis without damaging adjacent normal tissues, low concentrations of MB were preferential used to prevent possible toxic effects of residual photosensitizers remaining dyes [21].

It is well recognized that biofilm formation is initiated by bacterial adhesion, which in turn constitutes a three-dimensional structure and develops a mature entity [54]. Thus, it is indeed necessary to evaluate the number of bacteria and its remaining LPS on the Ti alloy surface after various treatments. When the Ti alloy sample was exposed to the bacterial suspension for 24 h, marked bacterial colonization occurred. On the contrary, the use of cationic MB alone can reduce the growth of bacteria, but from the results of CFU assay, there was no sign of effective eradication effect, as confirmed by an earlier report [55]. MB can attach the bacterial membranes of Gram-positive and Gram-negative bacteria by interaction with the anionic region on the bacterial cell wall [5]. In addition, MB may be toxic to some extent. It is reasonable to think that strong antibacterial activity often resulted in high cytotoxicity, which was in line with the results reported by Miyata [56]. MB alone has a concentration-dependent bactericidal activity [5,55], but it fails to completely eradicate bacteria. Therefore, MB-PACT was used instead of MB to eliminate bacteria and biofilms more effectively. Not surprisingly, the percentage reduction of the two viable bacteria in MB-PACT was significantly improved compared with MB alone. Kim et al. pointed out that when MB alone caused 53% of cell death, the use of 100 μg/mL MB with the Periowave diode laser can reduce 77% of *A. actinomycetemcomitans* attached on the SLA titanium surface [57]. The higher MB concentration in MB-PACT lead to higher bactericidal activity, which was consistent with the previous studies [18,58]. Another interesting study was to test the potential role of QTS as an antimicrobial adjuvant, which was believed to help prevent bacterial infection and adhesion on the surface of the implant. According to a previous study [35], the concentration-dependent bactericidal activity of QTS against the Gram-positive bacteria and Gram-negative bacteria examined is due to the production of reactive oxygen species (ROS). Moreover, cationic QTS may destroy the integrity of the biofilm and kill the microbial cells by piercing the surface of negatively charged microbial cells [37]. Results of SEM images corroborated the current findings of the bacteria count.

Since MB alone and QTS alone had a potent antimicrobial efficacy, it can be speculated that the combination of the two agents had an additive effect on eliminating bacteria. In fact, the cationic QTS + MB solution provided advantages by allowing the construction of a viscous photosensitizing platform as a multifunctional role including the MB-PDT efficacy and the inherent antibacterial ability of QTS. Camacho-Alonso et al. pointed out that the chitosan + MB-PACT group resulted in the lower CFU/mL count of Enterococcus faecalis compared with MB-PACT alone in experimentally infected root canals of extracted human teeth, but there was no significance between these groups [59]. Darabpour et al. reported that chitosan nanoparticles destroyed the biofilm structure of *Staphylococcus aureus* and *Pseudomonas aeruginosa*, which in turn made MB deeper and higher permeability, thereby enhancing the eradication efficacy of MB-PACT [60]. Carpio-Perochena et al. also found that the combination of carboxymethyl chitosan/rose bengal and PACT can reduce more viable bacteria than carboxymethyl chitosan alone [61]. Regarding the effect of cellulose, MC1 did not reveal an additional antibacterial efficacy from the comparison between the MB100-PACT and MC1+MB100-PACT groups. In short, compared with the same concentration of MC, QTS helped to improve the antibacterial efficacy.

In addition to inactivating pathogenic bacteria, it is also important to clean the LPS (the major cell wall component of Gram-negative bacteria) from the surface of the implant because the LPS remaining on the material surfaces may not only facilitate subsequent microbial adhesion [62] but also jeopardize the proliferation of connective tissue cells [63]. Biofilm formation on the surface of the implant can protect bacteria and promote the persistence of infection, which is difficult to completely avoid on SLA-pretreated implants [64]. In addition, biofilm makes antibiotic treatment insufficient to eradicating the infection. Thus, residual LPS analysis should be conducted to unveil the importance of QTS + MB-PACT. Contrary to the high amount of LPS on the contaminated surface, PACT treatment resulted in a significant reduction in LPS caused by *A. actinomycetemcomitans*. There was a positive correlation between the increase in MB concentration and the decrease in LPS, which was in full agreement with the previous study [5]. On the other hand, there was no significant difference in residual LPS and bacteriostatic ratio between MB100-PACT and MC1+MB-PACT. As mentioned earlier, it was due to the insufficient antibacterial ability of MC.

Regarding the QTS + MB-PACT modality, the more positive charge of QTS + MB would have a higher affinity for LPS [65], which may be the reason why QTS + MB100 was higher than MB100 in the eradication of LPS after photoactivation. Therefore, benefit can be expected to use this novel therapeutic modality to enhance the treatment efficiency of peri-implantitis. Interestingly, in the QTS+MB-PACT modality, QTS1 eliminated LPS more effective than QTS3. Although QTS adhesive had viscous ability, it can firmly bond MB onto the robust Ti alloy surface, but too high QTS concentration (such as 3 wt%) may prevent the laser penetration through MB due to the optical shielding or inhibit the MB penetration to the bacterial membrane. Generally, PS molecules or PS-carrying particles can be transferred into cells through transmembrane and along-membrane diffusion, by non-specific endocytosis or even pinocytosis, or can also be internalized through phagocytosis [44]. Therefore, under the appropriate QTS concentration, the effective retention of MB near the cell membrane will have better photobactericidal activity.

In order to achieve long-term success in clinical practice, Ti implants must support the growth of cells and tissues. The presence of bacteria could complicate the process of osseointegration. In this sense, it was indispensable for verifying whether the contaminated Ti alloy treated with various PACTs used in this study can maintain the osteoblast function as uncontaminated Ti alloy control. A concentration of 100 μg/mL MB is used in the therapy of peri-implantitis, such as commercial Periowave modality [57,66]. Almeida et al. found in a rat model of periodontal disease that 100 μg/mL MB-PDT can effectively reduce bone loss in the furcation region of the first molar [66]. Based on the current results of cytotoxicity and antibacterial efficacy, this concentration was used to evaluate the in vitro osteogenic activity using MC63 cells.

Cell attachment and proliferation are the initial phase of cell–material interaction [67]. The results of the current study clearly indicated that compared with MC1 + MB100-PACT and MB100-PACT methods, QTS1 + MB100-PACT enhanced the cell attachment and proliferation of MG63 osteoblasts. This may be because that antibacterial QTS synergistically reduced bacterial colonies and LPS amount on the disinfected surface, making it less harmful to cell growth [63,68]. Eick et al. found that the cell attachment of human alveolar osteoblasts on disinfected SLA Ti implant treated with effective PDT was equivalent to the Ti surface without bacterial biofilm exposure [68]. After the cells grow, osteoblasts will continue to differentiate and secrete ALP (an earlier differentiation biomarker), and then deposit calcium [69]. In fact, with increasing culture time, the ALP and calcium content of MG63 cells increased significantly. Similar to the trend of cell proliferation, the ALP expression response to the decontaminated samples showed the order of QTS1 + MB100-PACT > MC1 + MB100-PACT ≅ MB100-PACT. The biocompatibility of MC can be used to explain why there was difference in antibacterial efficacy and osteoblast function between MC1 + MB100-PACT and MB100-PACT.

For re-osseointegration of decontaminated implant, the ability of cells to produce mineralized matrix is important. As a result, on the surface of infected samples treated with QTS1 + MB100-PDT, the calcium deposits of MG63 osteoblast were significantly higher than those of MC + MB100-PACT and MB100-PACT. More importantly, although the disinfection was not fully conducted, its osseointegration was comparable to the uncontaminated Ti alloy control. This result can be explained as a large number of osteoblasts can coexist with a small number of bacteria and grow well [5,70]. As stated by Gristina [70], the fate of biomaterials is the competition between bacterial adhesion and biofilm growth and tissue integration, which depends on the number of existing bacteria. As mentioned earlier, it has been proven that QTS+MB-PACT can remarkably reduce bacterial colonies, which was beneficial to osteoblast function on the surface of the decontaminated implant. The osteogenesis-related increase with the increasing culture time can be used to interpret the point. In short, due to the synergistic effect of QTS and MB photoactivation, MB-conjugated QTS could perform the dual functions of enhancing bacterial elimination efficacy and improving osseointegration of Ti implant. This PACT modality based on the QTS and MB synergetic system may be a promising approach of killing bacteria.

## 4. Materials and Methods

### 4.1. Preparation of QTS

According to previous research [35], quaternary ammonium chitosan (QTS) was prepared using chitosan polysaccharide (Sigma-Aldrich, St. Louis, MO, USA). Briefly, the as-received chitosan polysaccharide was dissolved in acetic acid, and then acetic anhydride (Echo Chemicals, Miaoli, Taiwan) was added to the solution. The reaction was blocked with NaOH, and the mixture was dialyzed against water and freeze-dried to obtain water-soluble N-acetylated chitosan. Glycidyltrimethylammonium chloride (GTMAC; Tokyo Chemical, Tokyo, Japan) as a quaternizing agent was added to 2.5% N-acetylated chitosan in water and reacted for 8 h. The product was placed in cold acetone and kept in the refrigerator for 24 h. After discarding the acetone, the remaining sample was dissolved in methanol for 1 h, and then precipitated in a 4:1 acetone–ethanol solution, followed by filtration and drying at 60 °C to obtain QTS, which was stored in 4 °C refrigerator before use. The chemical structure of QTS has been determined in an earlier study [35].

### 4.2. Preparation of Solution

Three concentrations (50, 100, and 200 μg/mL) of methylene blue (Riedel-deHaen, Buffalo, NY, USA) were prepared in phosphate buffer solution (PBS) with a pH 7 [5] and stored in the dark at 4 °C for usage later. Three concentrations of 0.5, 1, and 3 w/v% QTS solutions were obtained by dissolving the powder in PBS. In addition, MB was also dissolved in different QTS concentrations in a beaker wrapped with aluminum foil to a final concentration of 100 μg/mL under thoroughly stirring for 1 h to homogenize the composition, which was called QTS + MB100 mixed solution. One percent methyl cellulose (MC; Showa, Tokyo, Japan) in PBS was also used as an adhesive and control, compared with QTS. For simplicity, the sample code “MB100” represented the use of 100 μg/mL MB solution, and “QTS1” was for 1% QTS.

### 4.3. L929 Cytotoxicity

The L929 cytotoxicity of various agent concentrations in Dulbecco’s modified Eagle medium (DMEM; HyClone, Logan, UT, USA) was conducted according to ISO 10993-5 standard by a MTT 3-(4,5-dimethylthiazol-2-yl)-2,5-diphenyltetrazolium bromide; Sigma-Aldrich) assay. The L929 cell suspension (10^4^ cells per well) was cultured in 24-well microplates under a 5% CO_2_ humidified atmosphere at 37 °C for 1 day, and then exchanged with DMEM containing test agents for a culture time of 1 and 10 min. DMEM alone was used as a negative control, whereas DMEM containing 10% dimethyl sulfoxide (DMSO; Sigma-Aldrich) was used as a positive control. Ultimately, microplates were read at 563 nm using a BioTek Epoch spectrophotometer (Winooski, VT, USA). The absorbance results were recorded for three independent measurements. The cell viability was normalized to the negative control in terms of absorbance.

### 4.4. Viscosity

The Discovery HR-2 rheometer (TA Instruments, New Castle, DE, USA) with parallel plate geometry was used to evaluate the viscosity (η) of PBS, MB100, MC1, and three QTS solutions at room temperature. The viscosity of the test solution was a function of the shear rate between 0.1 and 500 1/s, with ten data points per decade.

### 4.5. Preparation of Titanium Alloy

A 10 × 10 × 3 mm^3^ Ti6Al4V alloy disc (ASTM F136-84; Titanium Industries, Parsippany, NJ, USA) was used as the implant substrate and wet-ground with a 3M Wetordry 1200 grit SiC sandpaper (St. Paul, MN, USA). After sandblasting with 100 µm Al_2_O_3_ particles (Korox, Bego, Bremen, Germany) for 10 s, the disc was acid-etched in HCl/H_2_SO_4_/H_2_O (1:1:100) at 100 °C for 30 min to produce the SLA surface [64]. Through ultrasonically cleaning in acetone and ethanol for separate 20 min, the disc was rinsed in deionized water and then dried in an oven at 60 °C. Figure S1 schematically illustrated the experimental procedure including solution analyses, PDT steps, antibacterial evaluation, and osteoblast function examination.

### 4.6. Solution Flowability

To verify the retention ability of various agents on the implant surfaces, the MB100 photosensitizer solution with and without QTS or MC1 was dropped onto the surfaces of the Ti alloy plate with a 90-degree inclination angle. After 1 min, an image was taken by camera to observe the flowability of the test agent.

### 4.7. Bacteria Seeding

Gram-negative *Aggregatibacter actinomycetemcomitans* (*A. actinomycetemcomitans*; IDH 781) and Gram-positive *Streptococcus mutans* (*S. mutans*; ATCC 700610) bacteria were used as the bacterial species tested. The bacteria were cultured in the Bacto tryptic soy broth (Beckton Dickinson, Sparks, MD, USA) and grown to the optical density of about 1.0 at 600 nm detected by using a Beckman Coulter DU-640 spectrophotometer (Fullerton, CA, USA). They were diluted by broth to a density of 2 × 10^6^ colony-forming units (CFU)/mL. The SLA Ti alloy discs were randomly allocated to 24-well culture plates and then sterilized by soaking in a 75% ethanol solution and exposure to UV light overnight. After that, 1 mL of the bacteria suspension was inoculated onto the disc surface and cultured in an incubator at 37 °C for 1 day to attach. The contaminated samples were gently washed with PBS (pH 7.4) twice to remove non-adherent bacteria. The contaminated Ti alloy disc without any treatment was used as a control, while the MB, QTS, QTS+MB100, and MC1+MB100 groups with and without PACT treatment were assigned as the experimental groups.

### 4.8. Photodynamic Treatment

The contaminated disc sample was coated with 100 μL of test solution for 1 min of reaction and then washed by PBS. Afterwards, some samples were irradiated with a low-level AlGaInP diode laser (Aculas-HB, Konftec, New Taipei City, Taiwan) with an output diameter and 8 mm. The laser beam with maximum output power of 80 mW (4.8 J/cm^2^) was irradiated in a continuous mode at 660 nm for 1 min [58], and the irradiation distance was 10 mm at an incidence angle of 90°.

### 4.9. Antibacterial Efficacy

#### 4.9.1. Bacterial Counting

For bacterial counting, the conventional spread plate method was used to count the number of bacterial colonies distributed on the surface of the sample. After bacterial adhesion, contaminated sample washing and test solution spraying, some samples were treated by PACT, as described above. After that, 1 mL of PBS was added to each sample, and then the adhered bacteria were ultrasonically detached in a 150 W ultrasonic bath (DC150H, Taiwan Delta New Instrument Co. Ltd., New Taipei City, Taiwan) for 5 min at a frequency of 40 kHz. A 100 μL aliquot of the bacterial suspension was collected from each well, and four serial ten-fold dilutions were performed using PBS. Then, 100 μL of the bacterial dilution was spread on a 15 mL of Trypticase soy agar (Conda, Madrid, Spain) plate (9 cm in diameter) for 1 day of incubation at 37 °C. The total numbers of CFU in each dish were counted and the bacteriostatic ratio (%) was calculated as follows:

Bacteriostatic ratio (%) = (Ncontrol − Nexperiment)/Ncontrol × 100%,

where Ncontrol and Nexperiment are the number of bacterial colonies on contaminated Ti alloy control (CFU/sample) and experimental groups (CFU/sample), respectively [71]. Ten measurements were taken for each group.

#### 4.9.2. Bacterial Colony Observation

To further observe the changes of bacterial colonies after treatment, the contaminated Ti control and the disinfected groups of QTS1, MB100, MB100-PACT, QTS1 + MB100-PACT, and MC1 + MB100-PACT were viewed using a scanning electron microscopy (SEM; JEOL JSM-7800 F, Tokyo, Japan). The samples were washed two times with PBS and fixed in 4% paraformaldehyde (Sigma-Aldrich) for 20 min, then dehydrated for 20 min at each concentration using a graded ethanol series, mounted on a stub, and then coated with a gold layer.

#### 4.9.3. LPS Detection

After PDT treatment and PBS washing twice, the ToxinSensor chromogenic Limulus Amebocyte Lysate endotoxin assay kit (GenScript, Piscataway, NJ, USA) was used to detect the amount of LPS remaining on the sample surface [58]. A sterile Ti alloy sample was used a negative control, which a Ti alloy sample contaminated with A. actinomycetemcomitans was used as a positive control. The absorbance at 545 nm was the mean of five independent measurements using a BioTek Epoch plate reader (Winooski, VT, USA).

### 4.10. MG63 Cell Culture

MG63 human osteoblast-like cells (BCRC 60279; Hsinchu, Taiwan) were used to examine the effects of various PACT modalities on cell function of decontaminated Ti samples. The cells were suspended in DMEM supplemented with 10% fetal bovine serum (FBS) (Gibco, Langley, OK, USA) and 1% penicillin (10,000 U/mL)/streptomycin (10,000 μg/mL) solution (Gibco) in 5% CO_2_ at 37 °C. A cell suspension (10^4^ cells/well) in a 24-well plate was seeded on each sample, and an uncontaminated Ti alloy sample was used as a control.

#### 4.10.1. Cell Attachment

To observe cell morphology on the sample surface after initial 6 h incubation, the cells were washed three times with PBS and fixed in 2% glutaraldehyde (Wako, Tokyo, Japan) at 4 °C for 2 h. After dehydration in the graded ethanol series and drying using critical point dryer device (LADD 28000; Williston, VT, USA), the cell sample was coated with a gold layer and observed by SEM.

#### 4.10.2. Cell Proliferation

After 1, 3, and 7 days of incubation, cell proliferation was assayed using the MTT assay, according to the previous study [5]. The results were obtained through five independent measurements and reported in terms of absorbance at 563 nm detected with a BioTek Epoch plate reader.

#### 4.10.3. Alkaline Phosphatase Activity

To examine the early cell differentiation on the 7th and 14th days, an alkaline phosphatase (ALP) activity assay was conducted using the TRACP & ALP assay kit (Takara, Shiga, Japan) [72]. Five samples were used in each group, and the average was obtained at 405 nm absorbance using a BioTek Epoch plate reader.

#### 4.10.4. Calcium Deposit Quantification

Alizarin Red S staining method was used to quantify calcium deposits secreted by MG63 cells. After 7 and 14 days of incubation, the cells were washed with PBS and fixed in 4% paraformaldehyde at 4 °C for 10 min, and then stained in 0.5% Alizarin Red S (Sigma–Aldrich) in PBS for 10 min [72]. After that, the calcium mineral precipitate was extracted with a 10% cetylpyridinium chloride solution for 30 min. The absorbance of extract was detected at 562 nm using a BioTek Epoch plate reader. Five samples were used to obtain the average.

### 4.11. Statistical Analysis

A one-way analysis of variance (ANOVA) statistical analysis was used to assess significant differences between means. Duncan’s multiple comparisons were utilized to determine the significance of the standard deviation in the data between samples. Statistical data was analyzed using the SPSS 14.0 software for Windows (SPSS Inc., Chicago, IL, USA). In all cases, when the p-value was less than 0.05, the result was considered statistically significant.

## 5. Conclusions

From a viewpoint of clinical practice, there is a great need to treat peri-implantitis and gain re-osseointegration. Within the limit of this in vitro model, viscous QTS played two roles in MB-mediated PACT: one was a photosensitizer carrier, and the other is an antibacterial agent. This QTS adhesive effectively improved the retention of MB on the implant surface. Compared with MB-mediated PACT, QTS + MB-PACT can more effectively remove bacteria attached to the SLA-pretreated titanium surface and produce osseointegration. It is worth noting that QTS + MB modality was better than MC+MB modality in terms of antibacterial efficacy and osteogenesis. All in all, considering the compromise between cytotoxicity, retention ability, in vitro antibacterial efficacy, and osteogenesis into account, synergistic photoantimicrobial chemotherapy with 1% QTS and 100 μg/mL MB was a potential modality for the treatment of peri-implantitis, which was better than 1% MC+MB100. Further studies are required, including examination of multispecies biofilm model on screw-shaped implants and osteointegration in vivo, before QTS+MB-PACT can be used for clinical treatment.

## Data Availability

The data presented in this study are available in this article and supplementary materials.

## Supplementary Materials

The following are available online at https://www.mdpi.com/article/10.3390/ph14040346/s1. Figure S1: Schematic flowchart of the experimental process and purpose.
