# Supplementary material for: Synergistic Photoantimicrobial Chemotherapy of Methylene Blue-Encapsulated Chitosan on Biofilm-Contaminated Titanium
Source: Pharmaceuticals (Basel). 2021 Apr 9;14(4):346. doi: 10.3390/ph14040346 (PMC8069275; doi:10.3390/ph14040346)
Supplement: Supplementary file 1 [file pharmaceuticals-14-00346-s001.pdf]

Supporting information

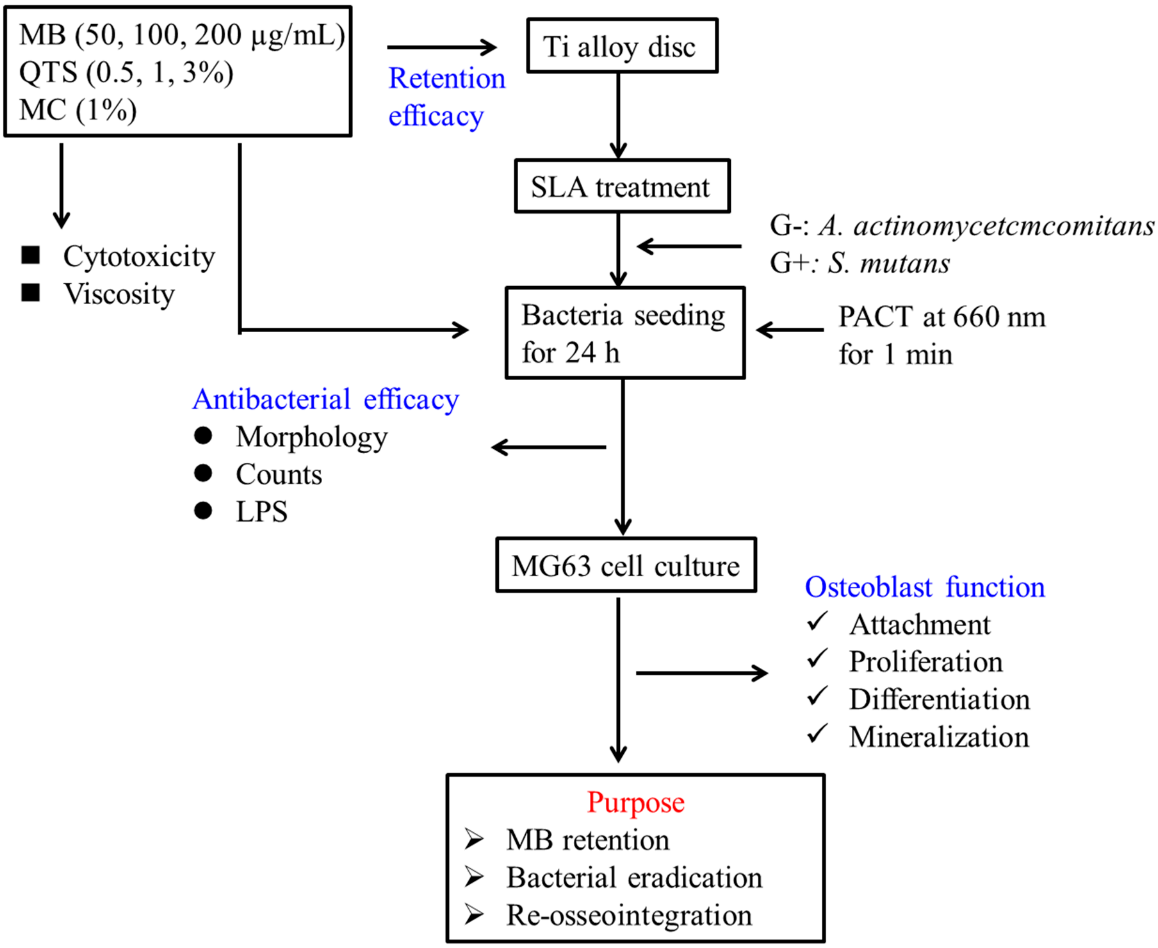

Figure S1. Schematic flowchart of the experimental process and purpose.
